# Encouraging people to set lower personal carbon budgets: anchoring is more effective than social reference groups

**DOI:** 10.3389/fpsyg.2025.1648500

**Published:** 2025-10-07

**Authors:** Sarah Lynn Flecke, Erika Aparicio, Eugene Malthouse

**Affiliations:** ^1^Department of Psychology, University of Innsbruck, Innsbruck, Austria; ^2^Department of Experimental Psychology, University College London (UCL), London, United Kingdom; ^3^Department of Psychology, University of Warwick, Coventry, United Kingdom; ^4^CogCo, The Cognition Company Group Ltd., London, United Kingdom; ^5^Centre for Decision Research and Experimental Economics, University of Nottingham, Nottingham, United Kingdom

**Keywords:** carbon footprint, carbon budget, anchoring, social reference group, pro-environmental behaviour, consumption

## Abstract

Enabling people to set personal carbon budgets may help them to track and reduce their carbon footprint over time. In this study, we investigated ways to encourage a representative sample of the UK population (*N* = 2,047) to reduce their carbon footprint by setting themselves a lower carbon budget. In an online experiment, we simulated a carbon footprint calculator based on personal spending and tested the effects of two carbon footprint anchors (low vs. high) and three social reference groups (people in the UK vs. customers at the same bank vs. people with similar expenditure) on the carbon budget set by participants. We found that providing a low anchor, independent of the corresponding social reference group, was significantly associated with setting a lower carbon budget. Setting a lower carbon budget was associated with greater motivation and self-reported willingness to change behaviour to adhere to the budget.

## 1 Introduction

As efforts to curb global carbon emissions intensify, addressing household emissions—driven largely by transport, food, and energy consumption—remains crucial. Household consumption accounts for over 60% of the global carbon footprint of emissions (Hertwich and Peters, 2009; Ivanova et al., 2016), and various strategies have been proposed to mitigate this impact. Among these, carbon taxes and carbon labelling are well-known policy measures aimed at reducing personal carbon emissions by making environmental costs more visible and financially impactful to consumers.

Personal carbon budgets, which encourage individuals to maintain emissions below a designated allowance, have also been considered as an approach to reduce personal carbon footprints (Wallace et al., 2010). Proposed by entities such as the UK’s Sustainable Development Commission since 2006 (Howell, 2012) and supported by figures like former German Chancellor Angela Merkel (Fawcett et al., 2009), personal carbon budgets would motivate individuals to manage their own emissions, just as financial budgets have been shown to motivate individuals to manage their finances (O’Neill et al., 2017). However, despite ongoing interest in the idea, personal carbon budgets have yet to be implemented on a large scale (Brock et al., 2022).

While no formal personal carbon budget policy currently exists, grassroots initiatives and private sector innovations have taken steps to support individuals interested in managing their carbon footprint. Voluntary groups, such as Carbon Rationing Action Groups (CRAGs), have encouraged people to set self-imposed carbon budgets, offering guidance and community support (Howell, 2012). More recently, several apps developed by private organizations provide tools for individuals to monitor and reduce their carbon footprints, as a supplement to broader policy measures. With these developments, personal carbon budgets represent both a promising tool for individual behaviour change now and a possible policy measure for the future.

It is important to note that carbon footprints are an accounting framework that visualizes the upstream and downstream impacts of consumption. Reducing one’s footprint does not automatically reduce emissions at the production side—for example, eating less meat lowers an individual’s footprint, but unless production volumes adjust, it does not immediately reduce overall emissions. Nevertheless, targeting footprints can still be useful, as it shapes demand and social norms that, when adopted at scale, put pressure on producers and policymakers to deliver emission reductions.

Given the potential of these private solutions to positively affect individual carbon-emitting behaviour—and the urgent need to avoid surpassing temperature thresholds associated with catastrophic climate tipping points—it is useful to understand how they may complement broader systemic efforts. While systemic policies, technological innovations, and regulatory frameworks are essential to meeting climate targets, individual actions can play an important role by shaping social norms and building engagement. This study explores a low-cost scalable solution to encouraging people to set ambitious personal carbon budgets, as part of this wider toolbox. Although research on pro-environmental behaviour has grown, few studies focus specifically on carbon budgeting as a means of personal action. This study addresses that gap, exploring effective strategies to encourage lower personal carbon budgets, fostering pro-environmental behaviour.

Many internal and external factors influence the extent to which we engage in pro-environmental behaviour, which can be broadly defined as behaviours we consciously engage in to minimise the negative impacts of our own actions on the natural and built world (Kollmuss and Agyeman, 2002). A systematic review of nudges for pro-environmental behaviour found that a range of interventions deployed in the decision setting can influence pro-environmental decisions. Across 160 experimental interventions, social influence and changes to the decision context (choice architecture) emerged as important influencers of pro-environmental behaviour (Byerly et al., 2018).

Though numerous changes to the choice architecture could be made, in the context of carbon budgets, anchoring presents particular promise. The anchoring effect describes the bias whereby seeing an initial numeric value influences people’s judgements and decisions thereafter (Kahneman and Tversky, 1979). The initial value shown biases the perception of a subsequent value up or down, depending on whether it is above or below the anchor. Different accounts for the persistence of anchoring effects have been offered, though in the context of carbon footprints, the “insufficient adjustment” explanation (Kahneman and Tversky, 1979) seems most relevant. In this view, the anchor provides a starting point for people to make their assessment about the given value. As they gather more information, they adjust away from the anchor until reaching a reasonable estimate (see, e.g., Epley and Gilovich, 2001, 2004).

The robustness of anchoring has been demonstrated in the context of legal judgements, purchasing decisions, negotiations, probability estimates, general knowledge, and real-world judgements [see, e.g., a review by Furnham and Boo (2011)]. Indeed, a recent meta-analysis finds a large effect size (Cohen’s *d* = 0.876) of the anchoring effect on real-world judgements (Schley and Weingarten, 2025). Within the environmental domain, several studies have found the anchoring effect to positively influence pro-environmental behaviours. For example, the portion of study participants willing to pay for air travel emissions was greater among individuals shown a higher priced flight, suggesting an anchoring effect for travel emissions taxes (Sonnenschein and Smedby, 2019). Other research on the use of anchors in the context of carbon emissions shows that individuals provided with a high anchor in combination with a normative message were willing to increase their total travel time for the benefit of a lower carbon emissions journey (Andersson et al., 2021; Bökman et al., 2021). Similarly, setting a higher default payment via a slider, which thus served as a price anchor in a simulated online flight booking system increased the amount paid for carbon offsetting (Székely et al., 2016).

Another intervention often used in decision settings is manipulating social influence, which involves presenting people with information about the behaviour of others. Introducing messages communicating social norms has been shown to be an effective tool for inducing pro-environmental behaviour [see, e.g., a review by Farrow et al. (2017)]. In their review, authors find that communicating descriptive norms—that which most people do—appears to be consistently effective within the environmental context (Farrow et al., 2017). Notably, such information has been successful when framed in such a way as not to emphasize undesirable behaviour (see, e.g., Cialdini et al., 2006; Schultz et al., 2007; Ferraro et al., 2011). In other words, when the targeted behaviour is undesirable (such as wasting a resource), framing a message negatively to show how much worse the target is performing relative to the social comparison group is a preferred strategy. A meta-analysis by Abrahamse and Steg (2013) found that social influence approaches are effective at promoting resource conservation, such as reducing water and gas use, or recycling. Effects of social comparison messages have even been shown to last over long time horizons: in a field study to reduce water usage, inclusion of a comparison of the household’s use to the median county household for the same period, coupled with an indication of the percentile in which the household fell, significantly improved water usage more than 2 years later, relative to households that received no social comparison message (Ferraro et al., 2011).

There is also evidence that normative messages and social comparisons are effective at improving behaviour within the specific context of reducing personal carbon footprints. US participants shown normative information regarding low-carbon consumption behaviour before making a purchasing decision were more likely to select a product with lower lifecycle emissions (Castro-Santa et al., 2023). Swedish participants shown a normative message about the maximum recommended individual yearly and weekly average CO2 emissions were willing to travel for longer, in order to reduce their CO2 footprint on the journey, but only if this was coupled with a high (ambitious) anchor (Bökman et al., 2021). These results are echoed by Andersson et al. (2021), who similarly found that a sample of UK participants were willing to accept a longer travel time with a lower carbon emission, relative to a shorter, higher footprint journey, only when they saw a normative message about yearly and weekly recommended emissions coupled with a high anchor. One explanation for these results is that people align their behaviour with that of others in order to meet social expectations (i.e., what they think others will do or demand of them; Gächter et al., 2025).

In the present pre-registered study^1^ we investigated ways to encourage a representative sample of the UK population (*N* = 2,047) to reduce their carbon footprint by setting a lower, more ambitious carbon budget. Previous research by Grinstein et al. (2018) shows that people’s quantitative understanding of the carbon footprint connected to their daily behaviours is quite poor, implying low carbon numeracy. Interventions that target people’s carbon footprints are, therefore, limited by the extent that individuals understand the scope of their own carbon emissions, and how these differ by activity. Using cues like reference points can provide further guidance and motivation for people to identify appropriate levels of carbon footprints and define reasonable carbon budgets for themselves.

The study was developed in partnership with Cogo,^2^ a company that allows individuals to track their carbon emissions through their mobile banking app. Cogo’s Personal Carbon Manager is integrated into several retail banking apps in the UK, allowing customers to see their estimated monthly carbon footprint based on their financial transactions. In addition, customers can set a monthly carbon budget for themselves and track how they are performing against it in real-time via their transactions.

Evidence from Andersson et al. (2021) and Bökman et al. (2021) suggests that in the context of personal carbon emissions, the combination of providing both extrinsic motivational information via a normative message and extrinsic motivation-neutral information via an anchor may be especially promising. Building on evidence within the context of personal carbon emissions, the present study experimentally tests whether presenting people with information about other peoples’ carbon footprints would encourage them to set a lower (more ambitious) monthly carbon budget, and whether the value shown (i.e., the anchor) mattered.

To achieve this, we simulated Cogo’s experience of carbon footprint tracking and then presented participants with information about the average carbon footprint of three social reference groups. The reference groups selected were based on the type of information available to Cogo via the Personal Carbon Manager, making it a realistic test case for what users may see in the future. Specifically, the reference groups were (1) people in the UK, (2) people with similar expenditure to you, and (3) customers at the same bank as you. We then varied the average carbon footprint that we told them pertained to these three groups: (1) 941 kg (low anchor) or (2) 1,058 kg (high anchor). These anchors were selected as they correspond to levels that were 20 and 10% lower than the monthly UK average, making them ambitious but realistically attainable. We also measured participants’ requests for a link to the Cogo website’s personal carbon manager so that they could set themselves a personal carbon budget as an indicator of pro-environmental behaviour. By clicking this link, they could obtain more detailed information about their own carbon footprint.

## 2 Methods

### 2.1 Participants

A representative sample of the UK adult general population (*N* = 2,047) was recruited via an opt-in panel provider^3^ and completed an online study in autumn of 2022. This slightly exceeded our pre-registered aim of recruiting 222 participants in each of our eight treatments. All participants took part under informed written consent and were compensated for their time by Bilendi. The survey adheres to ethical standards set by the European Society for Opinion and Marketing Research (ESOMAR) and the Austrian Standards certification (ISO/IEC 17065). Participants were screened for residing in the United Kingdom and for using a mobile banking app. The sample included 56.7% female participants and 42.9% male participants, and the mean age was 47.6 (SD = 13.6, median = 48). These demographics were balanced across our treatment groups (see Appendix Table 4 for further details).

### 2.2 Procedure

After screening and agreeing to participate in the study, participants were randomly assigned to one of 8 treatment conditions or a control. The study was conducted fully online, with data collected via Qualtrics.

Participants were initially asked a series of multiple-choice questions about their habits as an indicator of their carbon footprint. We presented participants with different example bank statements related to different purchase types (for further details including screenshots of these, please see section 2 of the Appendix). First, participants were shown three example bank statements showing food-related purchases. One statement had a high carbon footprint, one had a medium footprint, and one had a low footprint, and we asked them which statement most closely resembled their own spending habits in a typical month. We then followed this same process with statements showing purchases related to energy use and travel. We included these screens to more closely simulate the experience participants would have engaging with banking apps partnered with Cogo. It also provided us with estimates of their average carbon footprint expenditures. Note that we did not actively calculate participants’ carbon footprint in real-time, but rather presented all participants with an estimated monthly footprint of 1,176 kg, which was the reported UK average at the time (Kayser, 2023). This allowed for us to measure the effect of the treatments, while keeping the initial value (monthly footprint) constant for all participants. At the end of the study, we provided all participants the opportunity to gain a more accurate and personalized estimate via the Cogo website.

After presenting participants with their estimated carbon footprint of 1,176 kg, we introduced the concept of carbon budgets and then asked them to set a budget for themselves. At this stage, participants in the control condition received no further information other than their estimated average monthly footprint, while those in all other treatment conditions were presented with additional information about the carbon footprint of others, as summarised in Table 1. In this way, the experiment consisted of a between-subjects 2 × 3 factorial design. We additionally tested two further treatments as an exploratory extension, which we report on separately. The two experimental independent variables were the anchor and the social reference group. The anchor had two levels: (1) a high anchor (1,058 kg average monthly carbon footprint) and (2) a low anchor (941 kg average monthly carbon footprint). Note that a “high” anchor is actually a higher carbon footprint, but a less ambitious target, since the closer to zero a footprint, the fewer CO2 emissions are generated. The social reference group had three levels: (1) UK average, (2) people with similar expenditures, (3) people at the same bank.

**Table 1:** 2×3 factorial design.

Anchor	Social reference group		
High (1,058 kg)	UK Average	People with similar expenditure to you	Customers at the same bank as you
Low (941 kg)			

The table summarises our six treatment conditions with two anchor levels and three social reference group levels.

**Figure 1:**
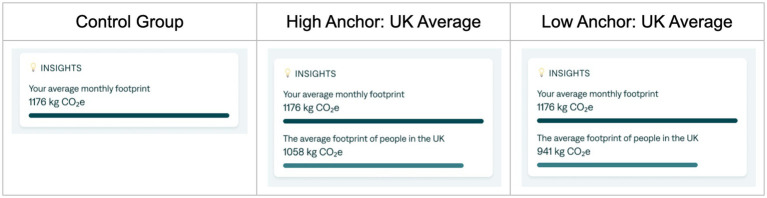
Carbon footprint estimate and anchor for control group and two treatment conditions.

Participants were shown a statement about their average monthly footprint with a visual bar representing the expenditure and, depending on the condition, a statement with information about others below it and a visually shorter bar (see Figure 1).

Participants were asked to set a monthly carbon budget in kg using a slider. The starting point of the slider depended on their condition. The control condition had the slider set to the estimated average monthly footprint of 1,176 kg, while the high anchor conditions had the slider set to 1,058 kg and the low anchor conditions had it set to 941 kg. These high and low anchors were 10 and 20% less than the average budget of 1,176 kg, respectively.

**Figure 2:**
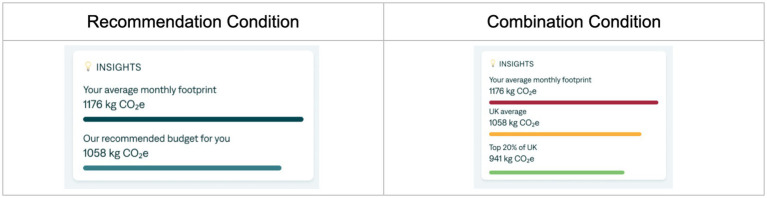
Additional exploratory conditions with high anchor recommendation and a combination of high and low anchor visual.

In addition to the discussed treatment conditions, we also tested the effect of two additional treatments as exploratory extensions: “Recommendation” (*N* = 215) and “Combination” (*N* = 238). In the Recommendation treatment, we tested the effect of stating a recommended carbon budget of 1,058 kg, equivalent to the high anchor value. In the Combination treatment, we tested the effect of showing both the high anchor and low anchor values as two separate reference groups in the same dashboard (see Figure 2).

Following the budget-setting, we asked participants a series of follow-up questions about their motivation to reduce their carbon footprint, their perception of their carbon footprint, their willingness to change their behaviour to reduce their footprint, and their interest in knowing about the impact of their spending. At the end of the study, we informed all participants that the carbon footprint we had calculated was estimated based on the UK average. Participants were asked if they would like to calculate a more accurate estimate of their own footprint. If so, they received a link to the Cogo website, where they could access a free app to calculate their carbon footprint and set an actual carbon budget. Requests for the link served as a measure of pro-environmental behaviour.

### 2.3 Hypotheses

Previous research demonstrates that anchoring can strongly influence numerical judgments, including in environmental contexts. In studies of carbon footprints, participants shown lower or more ambitious anchors set themselves more ambitious targets (Andersson et al., 2021; Bökman et al., 2021). Anchors are thought to work because people start from the presented value and adjust insufficiently away from it (Epley and Gilovich, 2004; Kahneman and Tversky, 1979). Based on this, we expected that participants exposed to a reference point would set lower budgets than those in a control condition, and that a lower anchor would produce more ambitious budgets than a higher anchor.

Social identity theory suggests that the relevance and proximity of a social comparison group influences how strongly people respond to normative cues (see, e.g., Lede et al., 2019), Previous work has shown that reduced psychological distance makes social norm messages more effective (Hallsworth et al., 2017), and meta-analytic evidence supports the role of social influence in promoting resource conservation (Abrahamse and Steg, 2013). We therefore expected that more specific or relatable groups, such as people with similar expenditure patterns, would elicit stronger effects than broader or less salient groups.

Feelings of moral obligation and personal responsibility can motivate pro-environmental action. For example, a multi-national survey found that three quarters of surveyed air travellers were willing to pay a carbon tax primarily due to feelings of moral obligation and personal responsibility to pay for their contribution to climate change (Brouwer et al., 2008). Normative information can also strengthen this sense of responsibility: recent evidence shows that social comparisons can trigger ascription of environmental responsibility, which in turn predicts pro-environmental behaviour (Ai and Rosenthal, 2024). In our study, referencing a social comparison group that performs better—this is, has a lower carbon footprint - may therefore increase participants’ sense of responsibility and willingness to obtain further information about reducing their own footprint. We thus expected participants in the treatment groups to be more likely to request a link to the Cogo website than those in the control, with the “similar expenditure” condition eliciting the highest likelihood.

We also planned pre-registered exploratory analyses examining whether the budgets participants set were associated with their attitudes—specifically, their motivation to reduce their carbon footprint, their self-perception of their footprint, and their willingness to change behaviour. We do not make directional predictions for these.

Hypotheses:

*H1*: Participants in treatment conditions will set lower carbon budgets than those in the control.

*H2*: Participants shown a lower anchor (941 kg) will set lower budgets than those shown a higher anchor (1,058 kg).

*H3*: Among reference groups, the “similar expenditure” condition will lead to the lowest budgets.

*H4*: Participants in treatments will be more likely than control to request the app link at the end of the study.

*H5*: The “similar expenditure” group will be the most likely to request the app link.

### 2.4 Data analysis

To test the first hypothesis, that the treatment conditions will affect lower carbon budget setting, we first pooled participants in all treatment conditions together. We then compared the carbon budgets they set for themselves with those set by participants in the control group with a linear regression model. For additional robustness, we performed a two-sided Mann–Whitney test (rather than a *t*-test) to account for the non-normal distribution of responses.

H2 and H3 dealt with the effect of social reference groups and anchors on personal carbon budgets, respectively. We tested these hypotheses together via a two-way ANOVA with the anchor and social reference group as independent variables and carbon budget as the dependent variable. In our pre-registration we stated that we would further investigate any effects with two-sided *t*-tests but instead we used Mann–Whitney tests since responses deviated significantly from a normal distribution.

H4 and H5, which both dealt with the effects of the treatment conditions on the likelihood to request further information via clicking the link at the end of the study, were tested with logistic regression models. For these models we coded participants who requested a link to the Cogo website as 1 and those who did not request the link as 0. For H4 (treatment conditions vs. control) we pooled all treatment participants and included this as the only independent variable. To test H5 (the effect of the social reference group), and the effect of the anchor, we conducted a logistic regression model with treatment as the independent variable and the control group as the reference category.

Lastly, according to our pre-registered exploratory analyses, we also investigated the relationship between participants’ carbon budgets and: (a) their motivation to reduce their carbon footprint; (b) their perception of their carbon footprint; (c) their willingness to change their behaviour in order to reduce their carbon footprint; and (d) their interest in knowing the carbon emissions associated with their current spending. To achieve this, we made participants’ responses numeric and calculated the Spearman’s rank correlation with their carbon budget.

## 3 Results

Our first finding was that participants in our treatments who were provided with a reference point set a lower carbon budget for themselves than participants in our control group (who were not provided with a reference point). For an overview of the main findings per condition, please see Table 2 for descriptive statistics below. A linear regression model indicated that participants across all six treatments set a statistically significantly lower carbon budget than those in the control group [−55 kg, (95% CI: −85 kg, −24 kg), *t*(1590) = −3.4, *p* < 0.001]. For additional robustness we conducted a Mann–Whitney test to compare the mean carbon budgets in the treatments (1,016 kg) and the control group (1,071 kg) which supported this conclusion (*W* = 182,784, *p* < 0.001, rank bi-serial correlation = −0.15). In summary, any reference point was better than none, providing evidence for H1.

**Table 2:** Descriptive statistics.

Treatment	*N*	Carbon budget	% taking action
Control	235	1,071	31.5
High Anchor / UK Average	224	1,053	29.9
High Anchor / Similar Expenditure	227	1,039	30.8
High Anchor / Same Bank	223	1,055	35.4
Low Anchor / UK Average	237	984	29.1
Low Anchor / Similar Expenditure	223	982	32.7
Low Anchor / Same Bank	222	982	41.4
Recommendation	216	1,057	32.9
Combination	240	1,004	32.9

The table shows the number of observations in each treatment, as well as the mean carbon budget that participants set, and the proportion of participants in each treatment who requested a link at the end of the study that would enable them to calculate their monthly carbon footprint.

**Figure 3:**
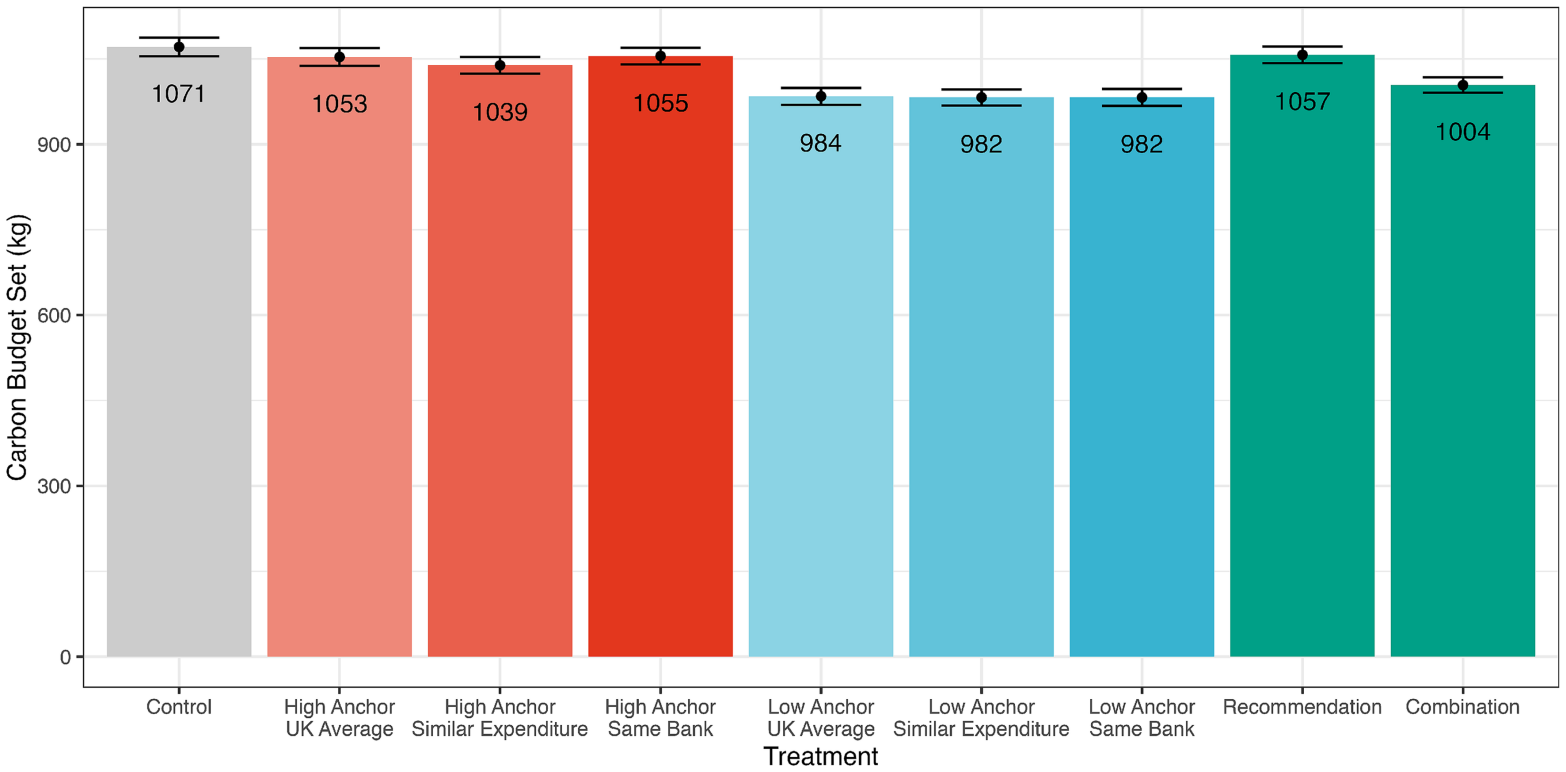
Carbon budgets by anchor and social reference group. The plot shows the mean carbon budgets set by participants in each treatment (error bars represent standard errors). Bars in red represent participants who were presented with a high anchor of 1058 kg. Bars in blue represent participants presented with a low anchor of 941 kg.

Our second finding was that the anchor we provided to participants influenced the carbon budget they set for themselves, whereas the social reference group information did not. A two-way ANOVA indicated a significant anchor effect [*F*(1,1350) = 30, *p* < 0.001, generalised *η^2^* = 0.02] but no social reference group effect [*F*(2,1350) = 0.2, *p* = 0.814, generalised *η^2^* = 0] or interaction between these between-subject variables [*F*(2,1350) = 0.2, *p* = 0.847, generalised *η^2^* = 0]. A post-hoc test (with a Holm correction for multiple hypothesis testing) showed that the low anchor resulted in a significant reduction in the carbon budget set [−66 kg, (95% CI: −90 kg, −42 kg), *t*(1350) = −5.5, *p* < 0.001]. A two-sided Mann–Whitney test also indicated that the average carbon budget of 983 kg set in the low anchor groups was significantly lower than the average carbon budget of 1,049 kg set in the high anchor groups (*W* = 282,661, *p* < 0.001, rank bi-serial correlation = −0.23). These findings (shown in Figure 3) provided support for H2 but not for H3 since the anchor, but not the social reference group, encouraged people to set a lower and more ambitious carbon budget.

**Figure 4:**
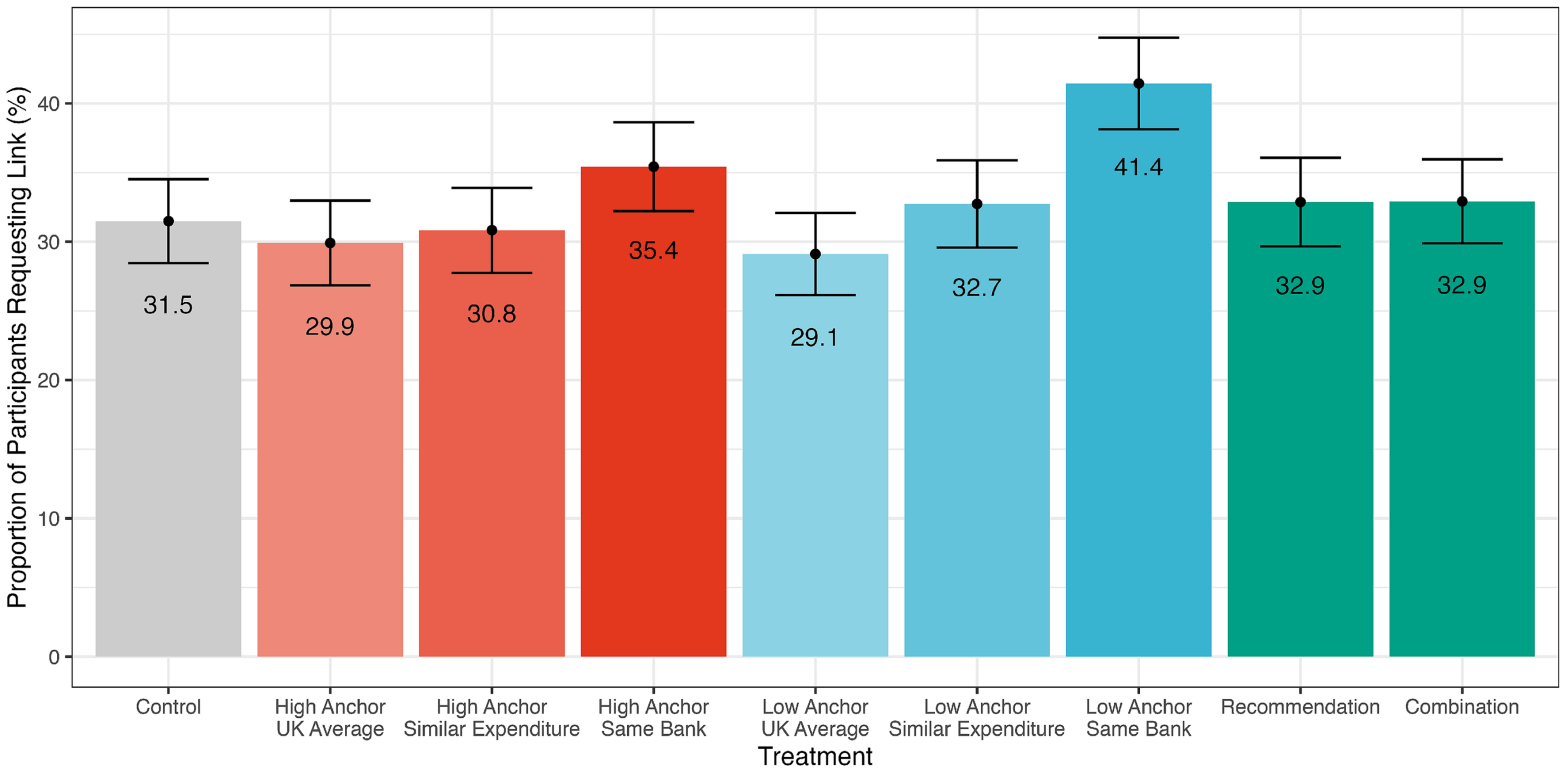
Pro-environmental behaviour between treatments. The plot shows the mean proportion (%) of participants in the control group and treatments who requested additional information about their carbon footprint (error bars represent standard errors). Low anchor same bank condition was significantly more likely than the control condition to request further information.

Next, we tested H4, H5, and the effect of the anchor on pro-environmental behaviour by examining our indicator of pro-environmental behaviour: the proportion of participants who requested an external link that would enable them to calculate their monthly carbon footprint (see Figure 4). For H4 we first pooled the treatments together (as we did when testing H1). We then conducted a logistic regression model, which indicated that participants in our treatments were no more likely to make this request than those in the control group (+0.08, [95% CI: −0.22, 0.38], *z*(1590) = 0.51, *p* = 0.61). A chi-squared test also indicated that the proportion of participants who requested a link was not significantly higher in the treatments vs. the control group (*χ^2^* (1) = 0.2, [95% CI: −0.08, 0.05], *p* = 0.663, Cohen’s *h* = 0.04). In summary, we did not find any evidence to support H4.

Similarly, we did not find evidence for the effects of anchors or social reference groups (H5) on the likelihood that participants requested a link at the end of the experiment. A logistic regression model revealed that only in the low anchor / same bank treatment was the proportion of participants requesting a link higher than in the control [+0.43, (95% CI: 0.05, 0.82), *z*(1590) = 2.2, *p* = 0.027] although this did not hold when applying a Holm correction for multiple hypothesis testing (*p* = 0.219). In other words, we did not find clear evidence that the anchor effect extended to our behavioural proxy (clicking the web link to further information). We acknowledge, however, that this measure has clear limitations, as decisions may have been influenced by privacy or trust. Recent work (Dowthwaite et al., 2024; Reyes-Cruz et al., 2024) shows that people may reject carbon budget apps due to concerns about accuracy, feasibility, fairness, and privacy. Our null results for the behavioural measure in the present study may, therefore, reflect such factors rather than a lack of motivation to act pro-environmentally.

Examining the additional “Recommendation” and “Combination” treatments, a simple linear regression model with treatment as the independent variable revealed no difference between carbon budgets set in the control group and the recommendation treatment (*t*(2038) = −0.66, *p* = 0.509). However, this same model showed that participants in the Combination treatment set themselves a significantly lower carbon budget (1,004 kg) than those in the control group (*t*(2038) = −3.27, *p* = 0.001, *p* (Holm correction) = 0.006). This was slightly higher than the budgets set by those in the low anchor conditions (983 kg), suggesting that when both anchors were present, the lower anchor (941 kg) had a stronger effect than the higher anchor shown (1,058 kg).

In a pre-registered exploratory analysis, we evaluated the relationship between the carbon budgets that participants set for themselves and their responses to a series of other questions relating to their attitudes about their carbon budget and footprint. See descriptive Table 3 for an overview.

**Table 3:** Descriptive statistics, additional questions.

Variable	Range	Mean	Median	Standard deviation
Carbon budget	588–1,764	1,025	1,006	225
Motivation to stick to budget	1–10	6.15	7	2.51
Perception of footprint	1–5	2.50	2	0.93
Willingness to change behaviour	1–5	3.50	4	1.01
Interest in knowing impact of spending	1–5	3.45	4	1.26
Understanding of carbon footprint	1–5	3.63	4	1.03

The table shows the range of possible responses, mean, median and SD for the set carbon budget and additional self-reported indicators. Self-reported motivation to stick to the budget uses a Likert scale of 1–10, where 1 = not motivated at all and 10 = strongly motivated. Participant’s perception of their carbon footprint is measured on a Likert Scale of 1–5, where 1 = I think I could do a lot better and 5 = I think that’s excellent. Willingness to change behaviour to lower one’s carbon footprint is measured on a Likert scale from 1–5 where 1 = Strongly disagree and 5 = Strongly agree. Interest in knowing about the carbon impact of one’s own spending is measured on Likert scale from 1–5, where 1 = Yes, definitely [would be interested] and 5 = No, definitely not. Understanding is measured on a Likert scale from 1–5, where 1 = Strongly disagree and 5 = Strongly agree with the statement “I fully understand what a carbon footprint is.”

**Figure 5:**
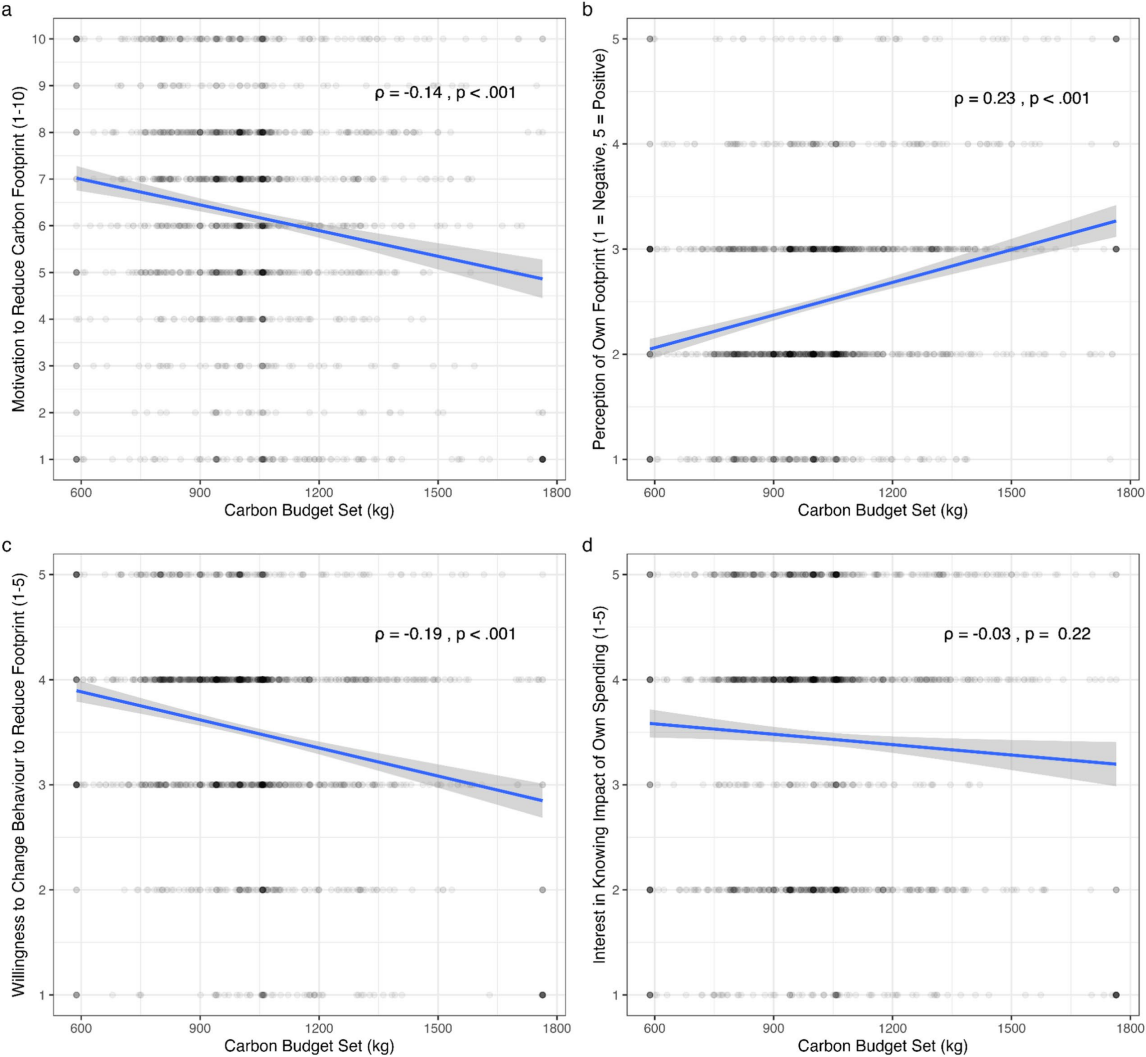
Attitudinal factors and carbon budgets. Plots show the relationship between participants’ carbon budgets and their motivation to adhere to the budget **(a)**, their perception of their carbon footprint **(b)**, their willingness to change their behaviour in order to reduce their footprint **(c)**, and their self-reported interest in knowing the impact of their own spending **(d)**.

We detected a statistically significant correlation between participants’ carbon budgets and their motivation to stick to their budget (*ρ* = −0.14, *p* < 0.001); their perception of their own carbon footprint (*ρ* = 0.23, *p* < 0.001); and willingness to change their behaviour to reduce their carbon footprint (*ρ* = −0.18, *p* < 0.001). In other words, the more negatively people perceived their carbon footprint, the lower the carbon budget they set for themselves. We further observed that the lower the carbon budget they set, the more motivated they were to stick to it, and the more willing they were to change their behaviour to reduce their footprint. We note that the direction of causality is not clear, and, while statistically significant, the correlations are weak. We did not detect any meaningful correlation between participants’ carbon budget and their interest in knowing the impact of their spending (see Figure 5).

We also examined participants’ self-reported comprehension of a carbon footprint, by asking them to what extent they agreed or disagreed with the statement, “I fully understand what a carbon footprint is.” 65.3% of participants agreed or strongly agreed with this statement, 19.0% were uncertain and 15.7% disagreed or strongly disagreed. Additionally, we also asked them to imagine why their footprint might have been higher at the end of the month than what they budgeted. 38% indicated it was likely because they had set an unrealistic budget that was too low, 30.8% indicated it was likely because they did not fully understand which actions contribute to a low carbon footprint, 19.0% state that is was likely because it was an unusual month with more high-carbon transactions than normal, 9.9% indicated it was likely because they were not motivated to stay within their budget and 2.2% indicated another reason.

As a non-pre-registered exploratory analysis, we also checked whether participant’s actual estimated footprint, as indicated by their pre-experimental selections of the bank statements that best reflected their typical spending patterns, were correlated with their perception of their own footprint. This provides a robustness check for pre-experimental differences in the sample. Based on the bank statements, 9.5% of participants had a relatively high carbon footprint, 53.5% had a medium carbon footprint, and 37% had a relatively low carbon footprint. We found a weak positive correlation between perception and actual estimated carbon footprint (*ρ* = 0.07, *p* < 0.001).

## 4 Discussion

Simulating a carbon footprint tracking tool embedded within a banking app (see Cogo), we investigated whether providing a carbon footprint reference point could influence the personal carbon budget that a representative sample of the UK general population (*N* = 2,047) set for themselves. Specifically, we tested whether seeing information about one of three social reference groups coupled with either a high (1,058 kg) or low (941 kg) carbon footprint anchor could encourage people to set more ambitious target budgets for themselves, relative to a control condition shown no reference point. Moreover, we examined how motivated participants were to adhere to their budgets, the perception of their own carbon footprints, the willingness to change their own carbon spending and their likelihood to take next steps by requesting a link to the Cogo website.

We found that overall, participants in the treatment conditions set lower (more ambitious) carbon budgets than those in the control condition, suggesting that any reference point was better than none. Furthermore, those shown a reference group with a lower carbon footprint (941 kg) set significantly lower budgets for themselves than those shown a reference group with a higher carbon footprint (1,058 kg). However, the social reference group provided—UK average, people with a similar expenditure, or people at the same bank – did not significantly affect the carbon budgets that study participants set. In other words, anchoring was more effective at eliciting lower carbon budgeting than the social reference group shown. In addition, our exploratory analysis revealed that our ‘Combination’ treatment also encouraged participants to set lower carbon budgets, suggesting that providing multiple anchors may also prove effective.

The absence of evidence for an effect of social reference group is somewhat surprising in light of the literature within the domain of environmental behaviour. The pooled effect of social influence approaches for reduction in resource consumption compared to a control group is small to medium (Abrahamse and Steg, 2013). Notably, results are heterogenous and, when accounting for publication bias, the effects are considerably smaller. Nevertheless, it is possible that in the present study, the selected reference groups were not deemed sufficiently relevant for eliciting a change in behaviour. Previous research on social norms has found that reduced psychological distance between the individual and the norm increases message salience and efficacy (Hallsworth et al., 2017). In other words, the more specific and relevant the reference group, the more relatable it becomes. We selected the reference groups to simulate more closely that which is currently possible within the research partner, Cogo’s platform, but more targeted reference groups like people living in the same region or neighbourhood, or people with similar incomes or family composition might have elicited stronger effects.

In light of previous research that carbon numeracy – understanding of the carbon footprint impacts of daily activities—is low (Grinstein et al., 2018), it is possible that limited familiarity with the relative meaning of 1 kg of CO2 emissions increased sensitivity to the anchor. For instance, prior to the introduction of the Euro in Germany, Germans were more strongly influenced by anchoring when making price estimates in Euros than after the currency was adopted, and familiarity was greater (Mussweiler and Englich, 2003). This is supported by work by Smith et al. (2013), who find evidence that anchoring effects are moderated by knowledge level. It is possible that the anchoring effects we observed may taper off as people become more familiar with their own carbon footprints and improve their carbon numeracy. Though we contextualized the carbon footprint information through the different questions about people’s purchases and habits (e.g., whether they follow a plant-based or vegetarian diet, whether they fly long-distance), when we asked participants indicate their level of agreement with the statement “I fully understand what a carbon footprint is,” 34.7% of the sample did not agree. Moreover, when we asked participants to imagine why the carbon footprint at the end of the month might have been higher than what they had budgeted, 31% of participants indicated that the most likely reason for this would have been that they did not fully understand which actions contribute to a low carbon footprint. These findings suggest that a relevant portion of participants had limited understanding of carbon footprints. This lack of understanding is noteworthy, as it may constrain the effectiveness of interventions that rely on carbon numeracy. That said, we suspect that if used regularly, the carbon footprint estimates coupled with the spending data in a banking app would serve to educate users over time as to what constitute higher and lower footprints. The persistence of the anchoring effect over time would, therefore, need to be investigated.

We also found that, pooled over all conditions, there was a significant correlation between setting a lower carbon budget and a higher self-reported level of motivation to adhere to it, as well as a greater willingness to change behaviour in order to reduce one’s footprint. This provides further support for the use of lower anchors; the lower anchor might affect not only the one-time setting of the budget, but could also boost enthusiasm for making lower carbon purchases. That said, it is also possible that people who were particularly motivated to stick to their budgets or more willing to change their behaviour, were also more willing to set lower carbon budgets. Further research would be required to verify the direction of causality here. Although all participants were initially provided with the same footprint estimate (1,176 kg), participant’s self-perception of this footprint varied. Interestingly, we observed a significant correlation between perception of one’s own carbon footprint and the carbon budget set. That is, those with a more negative perception of their own footprint also set lower (more ambitious) carbon budgets.

Interestingly, when we looked at people’s pre-treatment reported indicators of actual carbon footprint (typical monthly food, travel and clothing expenditures), participants who indicated having higher carbon footprints had a slightly more positive self-perception of their footprint, presenting a mismatch between perception and reality. This could imply that people with higher carbon footprints might not be fully aware of the extent of their environmental impact, which further speaks to the usefulness of providing contextualizing reference points. That said, the correlation, though significant, was weak, so we are cautious about over-interpreting this exploratory finding.

We also observed a behavioural outcome by providing participants an opportunity to request additional information about their carbon footprint. The proportion of participants in each group who requested this information served as an indicator of pro-environmental behaviour, beyond self-reporting. We found that only the low anchor + same bank condition increased the likelihood of making this request (41.4%) relative to the control (31.5%). Given that Cogo calculates carbon footprints by integrating with banking apps, it is possible that this effect was due to the salience of financial transactions during the experiment. We note, however, that this difference did not hold under multiple hypothesis testing. The anchor did not have an overall effect on behaviour; neither the high nor low anchor conditions were more likely to request additional information about their carbon footprints than the control condition. We also acknowledge that people may have avoided clicking the link for other reasons, including data privacy concerns or limited trust in the accuracy of carbon budgeting or estimation (Dowthwaite et al., 2024; Reyes-Cruz et al., 2024).

Placing additional questions between the budget-setting portion and the information request opportunity may have diffused participants’ engagement with the prompt, possibly reducing clicks. This separation was intended to make any observed requests for further information more robust, but it is possible that a greater portion of individuals may have selected further information, had this been available to them immediately following the budget-setting screen; that is, more closely following the time the treatment variation took place. We also note that we did not find that the level of green consumption (carbon-intensive spending on travel, food, or fashion) was correlated with likelihood to request further information, meaning that pre-experimental differences in participants do not explain the behavioural outcome.

This study has several limitations that should be pointed out. Although we found clear evidence that the anchor was effective, it remains unclear what the optimal level ought to be. Previous research has shown that moderate anchors generate stronger anchoring effects than extreme anchors (see, e.g., Chapman and Johnson, 1994; Mussweiler and Strack, 2001; Wegener et al., 2001). Thus, we would expect that setting the anchor too low may have a limited effect, as it becomes an unrealistic target. Future research could investigate the role of different anchors, using the same social reference group, in order to determine which level is most effective. Our setup ensured that the anchors shown were lower in all cases than the estimated footprint. In the context of energy reduction behaviour, boomerang effects have been observed when individuals learn they outperform the comparison (norm) group (Allcott and Rogers, 2014; Schultz et al., 2007). Similar effects might be expected, if the anchor had been set higher than the estimated individuals’ carbon footprint. Reducing the carbon footprint further among someone who is already living a low-carbon lifestyle is not the primary objective, but neither should we want them to increase their footprint as a result of such an intervention. The potential for such backfire effects could also be investigated further in future studies.

Our measure of behaviour, clicking a link to the personal carbon manager, also has limitations. We included it in order to capture more than stated behavioural intentions; following the link indicates going one additional step further to learn more or engage with the carbon footprint and budgeting tools. However, as noted above, there may have been non-environmental reasons keeping people from following the link. Privacy concerns or possibly being put off by a link to a non-governmental or non-educational website could have affected likelihood to click. To mitigate this, future work might include an alternative option, for example signing a personal climate pledge to reduce individual or household emissions.

A further limitation is that reductions in individual carbon footprints do not automatically translate into lower emissions at the production side and a focus on individual-level behaviour risks neglecting system-level approaches (Chater and Loewenstein, 2023). However, encouraging individuals to adopt lower budgets may still be valuable, as widespread changes in demand and social norms can, over time, create the conditions for systemic adjustments and emission reductions.

Finally, although we aimed to increase ecological validity of the present study by collecting data from a nationally representative sample and by simulating the experience and interface of an existing integrated carbon footprint calculator, the present study relies heavily on self-reported data. We capture behaviour as it pertains to participant interest to find out more information but cannot capture real spending behaviour as a result of budget-setting in the experiment. In future research it would be important to observe how the carbon budgets affect actual spending patterns, and whether these translate to lower-carbon purchases.

## 5 Conclusion

The present study demonstrates the robustness of the anchoring effect, also when applied to a novel context, like carbon budgets. We find evidence that the anchoring effect can help individuals set lower, more ambitious carbon budgets, suggesting a promising tool to foster behaviour change at scale. In our study, an 8% reduction was achieved in low-anchor conditions compared to the control, with participants in these conditions setting budgets 87 kg lower on average than those without an anchor intervention. If just 10% of the UK population adopted such a reduction target and adjusted their behaviours accordingly, this would equate to over 7 million tons of CO2 annually. Anchoring thus represents a promising, low-cost intervention that can be integrated into everyday tools such as banking apps to encourage more ambitious carbon budgeting. While not a standalone solution, it offers a practical way to engage individuals and normalize lower-carbon choices at scale.

## Data Availability

The raw data supporting the conclusions of this article will be made available by the authors, without undue reservation.

## Footnotes

^1^OSF pre-registration: https://osf.io/htp4n/?view_only=3b49a8934f7e4c3b943801f17bcc013d.

^2^ https://www.cogo.co/

^3^ https://www.bilendi.co.uk/

## References

[ref1] Abrahamse W. Steg L. (2013). Social influence approaches to encourage resource conservation: a meta-analysis. Glob. Environ. Chang. 23, 1773–1785. doi: 10.1016/j.gloenvcha.2013.07.029

[ref2] Ai P. Rosenthal S. (2024). The model of norm-regulated responsibility for proenvironmental behavior in the context of littering prevention. Sci. Rep. 14:9289. doi: 10.1038/s41598-024-60047-0, PMID: 38654095 PMC11039738

[ref3] Allcott H. Rogers T. (2014). The short-run and long-run effects of behavioral interventions: experimental evidence from energy conservation. Am. Econ. Rev. 104, 3003–3037. doi: 10.1257/aer.104.10.3003

[ref4] Andersson H. Ahonen-Jonnarth U. Holmgren M. Marsh J. E. Wallhagen M. Bökman F. (2021). What influences people’s Tradeoff decisions between CO2 emissions and travel time? An experiment with anchors and normative messages. Front. Psychol. 12:702398. doi: 10.3389/fpsyg.2021.702398, PMID: 34955942 PMC8699112

[ref5] Bökman F. Andersson H. Sörqvist P. Ahonen-Jonnarth U. (2021). The psychology of balancing gains and losses for self and the environment: evidence from a carbon emission versus travel time tradeoff task. J. Environ. Psychol. 74:101574. doi: 10.1016/j.jenvp.2021.101574

[ref6] Brock A. Kemp S. Williams I. D. (2022). Personal carbon budgets: a pestle review. Sustainability 14:9238. doi: 10.3390/su14159238

[ref7] Brouwer R. Brander L. Van Beukering P. (2008). “A convenient truth”: air travel passengers’ willingness to pay to offset their CO2 emissions. Clim. Chang. 90, 299–313. doi: 10.1007/s10584-008-9414-0

[ref8] Byerly H. Balmford A. Ferraro P. J. Hammond Wagner C. Palchak E. Polasky S.. (2018). Nudging pro-environmental behavior: evidence and opportunities. Front. Ecol. Environ. 16, 159–168. doi: 10.1002/fee.1777

[ref9] Castro-Santa J. Drews S. van den Bergh J. (2023). Nudging low-carbon consumption through advertising and social norms. J. Behav. Exp. Econ. 102:101956. doi: 10.1016/j.socec.2022.101956

[ref10] Chapman G. B. Johnson E. J. (1994). The limits of anchoring. J. Behav. Decis. Mak. 7, 223–242. doi: 10.1002/bdm.3960070402

[ref11] Chater N. Loewenstein G. (2023). The i-frame and the s-frame: how focusing on individual-level solutions has led behavioral public policy astray. Behav. Brain Sci. 46:e147. doi: 10.1017/S0140525X22002023, PMID: 36059098

[ref12] Cialdini R. B. Demaine L. J. Sagarin B. J. Barrett D. W. Rhoads K. Winter P. L. (2006). Managing social norms for persuasive impact. Soc. Influ. 1, 3–15. doi: 10.1080/15534510500181459

[ref13] Dowthwaite L. Reyes-Cruz G. Lu Y. Lisinska J. Craigon P. Piskopani A.-M.. (2024). Technology for Environmental Policy: exploring perceptions, values, and Trust in a Citizen Carbon Budget app. Proceedings of the Second International Symposium on Trustworthy Autonomous Systems, 1–13.

[ref14] Epley N. Gilovich T. (2001). Putting adjustment back in the anchoring and adjustment heuristic: differential processing of self-generated and experimenter-provided anchors. Psychol. Sci. 12, 391–396. doi: 10.1111/1467-9280.00372, PMID: 11554672

[ref15] Epley N. Gilovich T. (2004). Are Adjustments Insufficient? Personal. Soc. Psychol. Bull. 30, 447–460. doi: 10.1177/0146167203261889, PMID: 15070474

[ref16] Farrow K. Grolleau G. Ibanez L. (2017). Social norms and pro-environmental behavior: a review of the evidence. Ecol. Econ. 140, 1–13. doi: 10.1016/j.ecolecon.2017.04.017

[ref17] Fawcett T. Hvelplund F. Meyer N. (2009). “Making it personal: per capita carbon allowances” in Generating electricity in a carbon-constrained World, 87–107. Elsevier. doi: 10.1016/B978-1-85617-655-2.00004-3

[ref18] Ferraro P. J. Miranda J. J. Price M. K. (2011). The persistence of treatment effects with norm-based policy instruments: evidence from a randomized environmental policy experiment. Am. Econ. Rev. 101, 318–322. doi: 10.1257/aer.101.3.318

[ref19] Furnham A. Boo H. C. (2011). A literature review of the anchoring effect. J. Socio-Econ. 40, 35–42. doi: 10.1016/j.socec.2010.10.008

[ref800] Gächter S. Molleman L. Nosenzo D. (2025). Why people follow rules. Nature Human Behaviour, 1–13. 10.1038/s41562-025-02196-4 PMC12283409 40419800

[ref20] Grinstein A. Kodra E. Chen S. Sheldon S. Zik O. (2018). Carbon innumeracy. PLoS One 13:e0196282. doi: 10.1371/journal.pone.0196282, PMID: 29723206 PMC5933710

[ref21] Hallsworth M. List J. A. Metcalfe R. D. Vlaev I. (2017). The behavioralist as tax collector: using natural field experiments to enhance tax compliance. J. Public Econ. 148, 14–31. doi: 10.1016/j.jpubeco.2017.02.003

[ref22] Hertwich E. G. Peters G. P. (2009). Carbon footprint of nations: a global, trade-linked analysis. Environ. Sci. Technol. 43, 6414–6420. doi: 10.1021/es803496a, PMID: 19746745

[ref23] Howell R. A. (2012). Living with a carbon allowance: the experiences of carbon rationing action groups and implications for policy. Energy Policy 41, 250–258. doi: 10.1016/j.enpol.2011.10.044

[ref24] Ivanova D. Stadler K. Steen-Olsen K. Wood R. Vita G. Tukker A.. (2016). Environmental impact assessment of household consumption. J. Ind. Ecol. 20, 526–536. doi: 10.1111/jiec.12371

[ref25] Kahneman D. Tversky A. (1979). Prospect theory: an analysis of decision under risk. Econometrica 47:263. doi: 10.2307/1914185

[ref001] Kayser B. (2023). “What Is the Average Carbon Footprint per Person in the West?” Pawprint Eco Companion’.

[ref26] Kollmuss A. Agyeman J. (2002). Mind the gap: why do people act environmentally and what are the barriers to pro-environmental behavior? Environ. Educ. Res. 8, 239–260. doi: 10.1080/13504620220145401

[ref27] Lede E. Meleady R. Seger C. R. (2019). Optimizing the influence of social norms interventions: applying social identity insights to motivate residential water conservation. J. Environ. Psychol. 62, 105–114. doi: 10.1016/j.jenvp.2019.02.011

[ref28] Mussweiler T. Englich B. (2003). Adapting to the euro: evidence from bias reduction. J. Econ. Psychol. 24, 285–292. doi: 10.1016/S0167-4870(03)00015-1

[ref29] Mussweiler T. Strack F. (2001). Considering the impossible: explaining the effects of implausible anchors. Soc. Cogn. 19, 145–160. doi: 10.1521/soco.19.2.145.20705

[ref30] O’Neill B. Xiao J. J. Ensle K. (2017). Positive health and financial practices: does budgeting make a difference? J. Fam. Consum. Sci. 109, 27–36. doi: 10.14307/JFCS109.2.27

[ref31] Reyes-Cruz G. Craigon P. Piskopani A.-M. Dowthwaite L. Lu Y. Lisinska J.. (2024). “Like rearranging deck chairs on the titanic”? Feasibility, fairness, and ethical concerns of a citizen carbon budget for reducing CO2 emissions. PP. 267–78 in Proceedings of the 2024 ACM Conference on Fairness, Accountability, and Transparency, FAccT ’24. New York, NY, USA: Association for Computing Machinery.

[ref32] Schley D. Weingarten E. (2025). 50 Years of Anchoring: A Meta-Analysis and Meta-Study of Anchoring Effects (SSRN Scholarly Paper No. 5114456). Social Science Research Network. doi: 10.2139/ssrn.5114456

[ref33] Schultz P. W. Nolan J. M. Cialdini R. B. Goldstein N. J. Griskevicius V. (2007). The constructive, destructive, and reconstructive power of social norms. Psychol. Sci. 18, 429–434. doi: 10.1111/j.1467-9280.2007.01917.x, PMID: 17576283

[ref34] Smith A. R. Windschitl P. D. Bruchmann K. (2013). Knowledge matters: anchoring effects are moderated by knowledge level. Eur. J. Soc. Psychol. 43, 97–108. doi: 10.1002/ejsp.1921

[ref35] Sonnenschein J. Smedby N. (2019). Designing air ticket taxes for climate change mitigation: insights from a Swedish valuation study. Clim. Pol. 19, 651–663. doi: 10.1080/14693062.2018.1547678

[ref36] Székely N. Weinmann M. Brocke J. (2016). Nudging People to Pay CO2 offsets—the effect of Anchors in Flight Booking Processes. European Conference on Information Systems. Available online at: https://www.semanticscholar.org/paper/Nudging-People-to-Pay-CO2-offsets-the-effect-of-in-Sz%C3%A9kely-Weinmann/54dc487fea7e754107f501d1ea299a87b071b351

[ref37] Wallace A. Irvine K. Wright A. Fleming P. (2010). Public attitudes to personal carbon allowances: findings from a mixed-method study. Clim. Pol. 10, 385–409. doi: 10.3763/cpol.2009.0040

[ref38] Wegener D. T. Petty R. E. Detweiler-Bedell B. T. Jarvis W. B. (2001). Implications of attitude change theories for numerical anchoring: anchor plausibility and the limits of anchor effectiveness. J. Exp. Soc. Psychol. 37, 62–69. doi: 10.1006/jesp.2000.1431

