# Supplementary material for: Encouraging people to set lower personal carbon budgets: anchoring is more effective than social reference groups
Source: Front Psychol. 2025 Oct 7;16:1648500. doi: 10.3389/fpsyg.2025.1648500 (PMC12537360; doi:10.3389/fpsyg.2025.1648500)
Supplement: Supplementary file 1 [file Data_Sheet_1.pdf]

Appendix for

Encouraging people to set lower personal carbon budgets: anchoring is more effective than social reference groups

1. Additional Tables and Figures

Treatment	Average Age	SD	Median Age	Female	Male	N
Control	48.4	14.1	49	58.3%	41.3%	235
UK, High Anchor	48.5	13.6	48	54.9%	44.2%	224
Similar Expenditure, High Anchor	47.5	13.5	47	55.5%	44.1%	227
Same Bank, High Anchor	47.6	13.9	48	61.0%	39.0%	223
UK, Low Anchor	47.0	12.9	47	56.1%	43.9%	237
Similar Expenditure, Low Anchor	46.6	13.7	46	60.5%	39.5%	223
Same Bank, Low Anchor	47.6	13.9	49	51.4%	48.2%	222
Recommendation	46.4	12.7	46	57.9%	41.7%	216
Combination	47.7	13.0	47	57.1%	42.1%	240

Table 4. Demographics by Treatment Group

Includes control and 6 treatment conditions, plus two exploratory treatments

Anchor	Reference Group	Coefficient Estimate	Standard Error	95% Confidence Intervals	*t* value	*p* value
	Intercept (Control)	1070.91	14.77	1042.3, 1099.5	72.51	< .001
High (1,058 kg)	UK Average	-17.64	21.14	-58.5, 23.3	-0.83	0.404
	Similar Expenditure	-32.22	21.07	-73.0, 8.5	-1.53	0.126
	Same Bank	-14.96	21.14	-56.8, 25.0	-0.71	0.479
Low (941 kg)	UK Average	-86.73	20.84	-127.1, -46.4	-4.16	< .001
	Similar Expenditure	-88.67	21.16	-129.6, -47.7	-4.19	< .001
	Same Bank	-88.65	21.19	130.1, -47.2	-4.18	< .001

Table 5. Carbon Budgets Between Treatments

Summary of the output of a linear regression model where carbon budget is the dependent variable and treatment is the predictor variable.

2. App Screens Presented to Participants

The following screenshots present simulated banking app transaction history screens from the past month, which participants viewed during the study. Participants were asked to select the transaction screen that most resembled their own spending. These were presented in randomized order within each category. For each category, we included one high, one medium, and one low-carbon expenditure (this was not stated to participants). For the full set of instructions and questions, please see the attached Survey in the supplementary materials.

Transport-related transactions

Statement 1

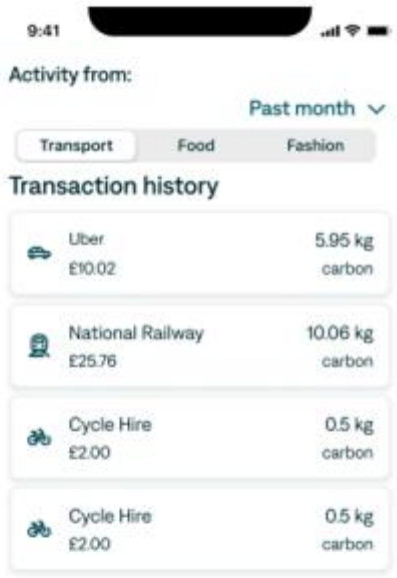

Statement 2

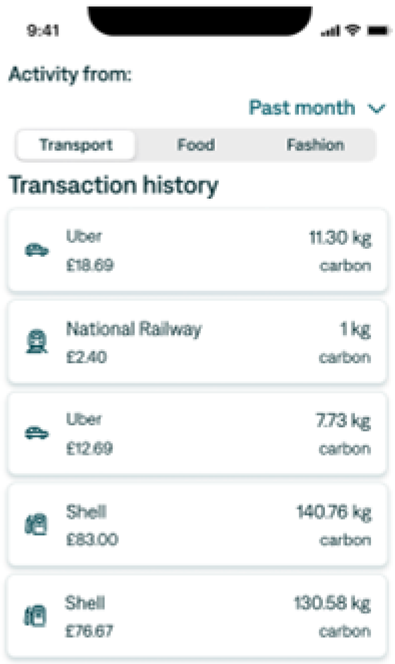

Statement 3

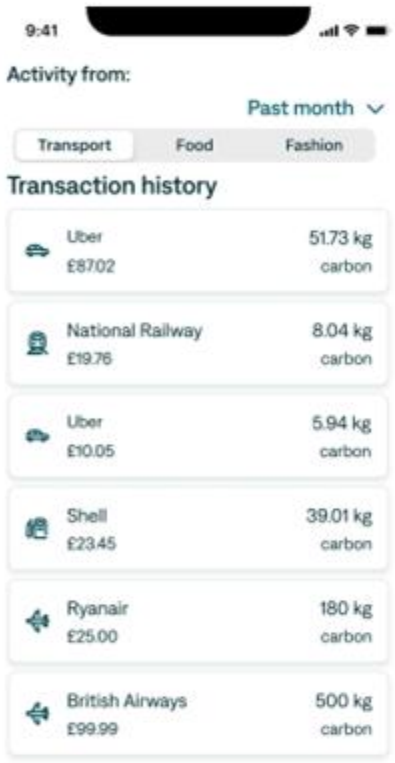

Food-related transactions

Statement 1

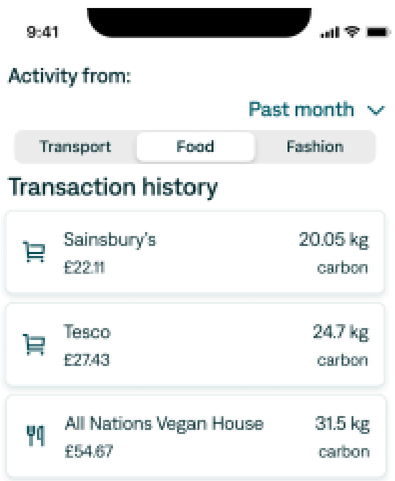

Statement 2

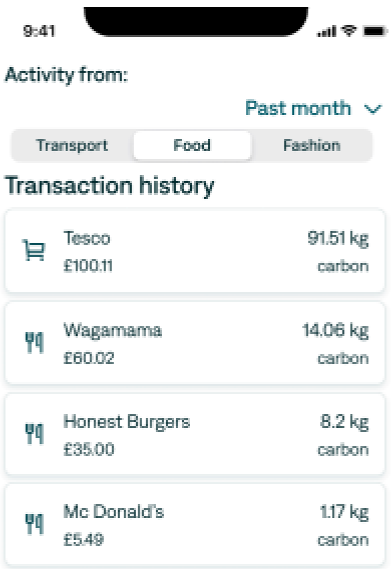

Statement 3

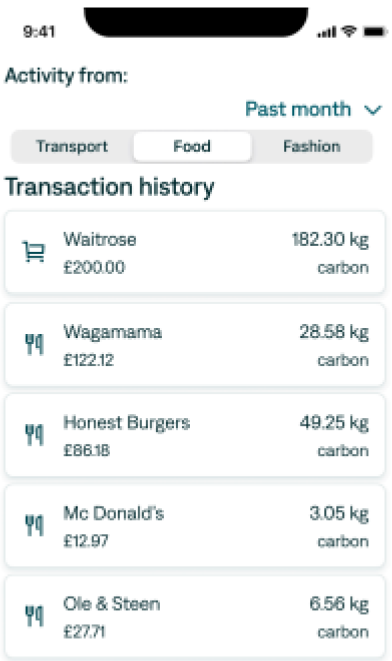

Fashion-related transactions[1]

Statement 1

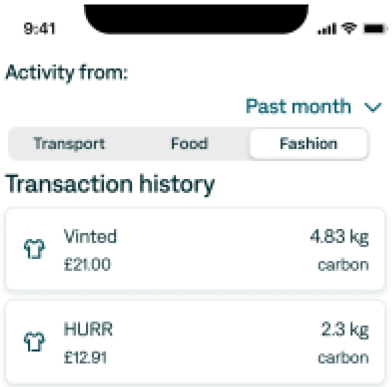

Statement 2

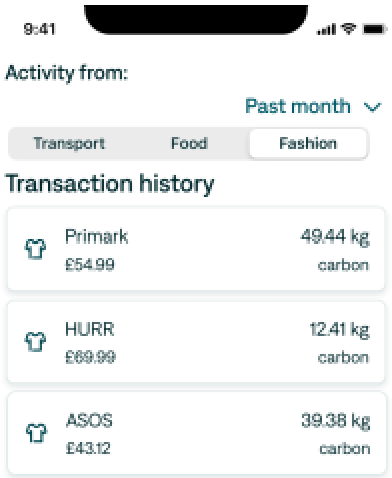

Statement 3

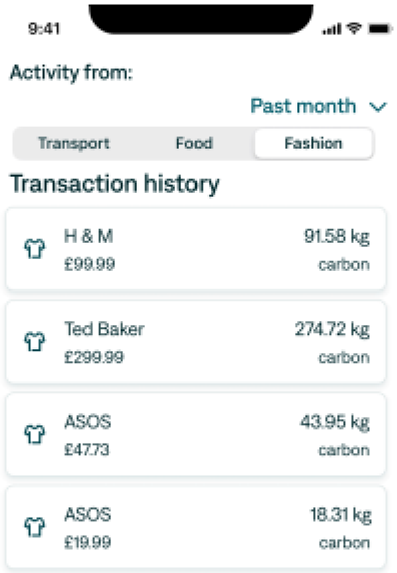

[1] Note that Vinted and HURR are online platforms for circular fashion (second-hand, wardrobe rentals)
